# Characterization of CYPs and UGTs Involved in Human Liver Microsomal Metabolism of Osthenol

**DOI:** 10.3390/pharmaceutics10030141

**Published:** 2018-08-30

**Authors:** Pil Joung Cho, Sanjita Paudel, Doohyun Lee, Yun Ji Jin, GeunHyung Jo, Tae Cheon Jeong, Sangkyu Lee, Taeho Lee

**Affiliations:** 1 BK21 Plus KNU Multi-Omics-based Creative Drug Research Team, College of Pharmacy, Research Institute of Pharmaceutical Sciences, Kyungpook National University, Daegu 41566, Korea; whvlfwjd@naver.com (P.J.C.); sanjitapdl99@gmail.com (S.P.); newkiy@hanmail.net (D.L.); yundzzang@naver.com (Y.J.J.); cgh0605@naver.com (G.J.); 2 College of Pharmacy, Yeungnam University, Gyeongsan 38541, Korea; taecheon@ynu.ac.kr

**Keywords:** Osthenol, CYP, UGT, human liver microsomes, glucuronidation

## Abstract

Osthenol is a prenylated coumarin isolated from the root of *Angelica koreana* and *Angelica dahurica*, and is an *O*-demethylated metabolite of osthole in vivo. Its various pharmacological effects have been reported previously. The metabolic pathway of osthenol was partially confirmed in rat osthole studies, and 11 metabolic products were identified in rat urine. However, the metabolic pathway of osthenol in human liver microsomes (HLM) has not been reported. In this study, we elucidated the structure of generated metabolites using a high-resolution quadrupole-orbitrap mass spectrometer (HR-MS/MS) and characterized the major human cytochrome P450 (CYP) and uridine 5′-diphospho-glucuronosyltransferase (UGT) isozymes involved in osthenol metabolism in human liver microsomes (HLMs). We identified seven metabolites (M1-M7) in HLMs after incubation in the presence of nicotinamide adenine dinucleotide phosphate (NADPH) and uridine 5′-diphosphoglucuronic acid (UDPGA). As a result, we demonstrated that osthenol is metabolized to five mono-hydroxyl metabolites (M1-M5) by CYP2D6, 1A2, and 3A4, respectively, a 7*-O*-glucuronide conjugate (M6) by UGT1A9, and a hydroxyl-glucuronide (M7) from M5 by UGT1A3 in HLMs. We also found that glucuronidation is the dominant metabolic pathway of osthenol in HLMs.

## 1. Introduction

**Figure 1:**
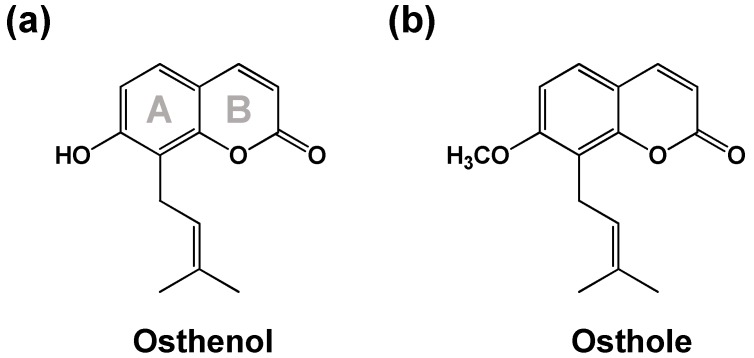
Chemical structures of osthenol (**a**) and osthole (**b**).

Osthenol (7-hydroxy-8-(3-methyl-2-butenyl)-2H-1-benzopyran-2-one, Figure 1a) is a prenylated coumarin isolated from the root of *Angelica koreana* and *Angelica dahurica* [1,2]. Its intermediate is a C8-prenylated derivative of umbelliferone, which is converted to an angelicin by angelicin and columbianetin synthase in the furanocoumarin biosynthetic pathway [3]. In previous studies, osthenol has shown anti-tumor, anti-fungal, anti-bacterial, and anti-inflammatory pharmacological effects. Osthenol showed the most potent inhibitory activity with only weak cytotoxicity on Raji cells, and mouse skin tumor promotion [4]. In addition, osthenol has strong antibacterial and antifungal activities against Gram-positive bacteria, as well as *Candida albicans* and *Aspergillus fumigatus* [5,6]. For anti-inflammatory effects, osthenol was shown to inhibit 5-lipoxygenase and cyclooxygenase in vitro [7].

The diverse pharmacological effects of osthenol have been reported, and it was assumed that osthenol, as the major metabolite of osthole (osthol, 7-methoxy-8-(3-methyl-2-butenyl)-2H-1-benzopyran-2-one, Figure 1b) in vivo, was involved in physiological effects of osthole, a natural coumarin found in the traditional herb Cnidii Fructus [8,9]. Osthole can convert to osthenol by *O*-demethylation, which was identified as phase I and phase II metabolites in rat urine after 40 mg/kg of osthole oral administration [10]. Nevertheless, the metabolic pathway of osthenol in humans still remains unclear. The aim of the present study was to investigate the metabolism of osthenol in human liver microsomes. We elucidated the structure of the generated metabolites using a high-resolution quadrupole-orbitrap mass spectrometer (HR-MS/MS) and characterized the major human cytochrome P450 (CYP) and UDP-glucuronosyltransferase (UGT) isozymes involved in osthenol metabolism.

## 2. Materials and Methods 

### 2.1. Materials

Osthenol (purity > 99%) was synthesized from 8-allyl-7-hydroxy-2H-chromen-2-one [11]. Pooled human liver microsomes (HLMs, UltraPool^TM^ HLM 50^®^), human recombinant cDNA-expressed CYP isoforms, and cDNA-expressed UDP-glucuronosyltransferase (UGT, Supersomes^TM^) were obtained from Corning Gentest (Woburn, MA, USA). Glucose 6-phosphate, glucose 6-phosphate dehydrogenase, uridine 5′-diphosphoglucuronic acid (UDPGA), β-glucuronidase, alamethicin, and 1,4-saccharolactone were purchased from Sigma-Aldrich (St. Louis, MO, USA). β-Nicotinamide adenine dinucleotide phosphate reduced form (β-NADPH) was purchased from Oriental Yeast Co. (Tokyo, Japan). All other chemicals were of analytical grade and used as received.

### 2.2. Identification of Osthenol Metabolites in Human Liver Microsomes

For Phase I metabolism, 20 µM osthenol was combined with 1 mg/mL pooled HLMs at 37 °C for 60 min with reduced nicotinamide adenine dinucleotide phosphate (β-NADPH)-regenerating system (NGS) in a 200 µL reaction volume in the presence of 0.1 M potassium phosphate buffer (Kpi, pH 7.4). To identify Phase II metabolites, 1 mg/mL HLM was treated with 0.5 mg/mL alamethicin and kept on ice for 15 min to activate the enzymes; then, 20 µM osthenol along with 0.1 M Kpi buffer (pH 7.4) was added to the mixture and incubated at 37 °C for 5 min. The reaction was initiated by the addition of 10 mM UDPGA and NGS solution and incubated for 60 min at 37 °C. The reaction was terminated by the addition of 400 µL 100% acetonitrile (ACN) mixed thoroughly and centrifuged at 13,000 rpm for 10 min. A 550 µL aliquot of the supernatant was transferred to a new tube and dried using vacuum concentrator. The dried sample was dissolved in 100 µL 20% MeOH containing 0.1% formic acid, and 10 µL of this mixture was injected onto a C18 column for liquid chromatograph tandem-mass spectrometer (LC-MS/MS) analysis.

### 2.3. Metabolism of Osthenol in Human Recombinant cDNA-Expressed CYP and UGT Isoforms

The reaction mixture consisted of 5 pmoles of human recombinant cDNA-expressed CYP isoforms (CYP1A2, 2B6, 2C8, 2C9, 2C19, 2D6, 2E1, 3A4, and 3A5) and 20 µM osthenol with NGS system in a 200 µL reaction volume in the presence of 0.1 M Kpi buffer (pH 7.4). Human recombinant UGT isoforms (UGT1A1, 1A3, 1A4, 1A6, 1A9, and 2B7, 50 µg/mL) were used for the analysis of the metabolites of UGT isoforms instead of human liver microsomes. Osthenol (10 µM) was pre-incubated in a 200 µL reaction mixture containing 0.1 mg/mL human recombinant UGT isoforms and 0.5 mg/mL alamethicin at 37 °C for 5 min, then and kept on ice for 15 min to activate the enzymes. Then, 0.1 M Kpi buffer (pH 7.4) was added to the mixture and incubated at 37 °C for 5 min. The reaction was initiated by the addition of 10 mM UDPGA and NGS solution and incubated for 60 min at 37 °C. The reaction was terminated by the addition of 400 µL 100% ACN, and mixed thoroughly. After incubation, the mixtures were centrifuged at 13,000 rpm for 10 min, and then 550 µL of supernatant was transferred to a new tube. The solvent was completely evaporated using a vacuum concentrator. The residue was dissolved in 100 µL 20% MeOH containing 0.1% formic acid. The analysis of osthenol metabolites was as described in the above section.

### 2.4. Reaction Phenotyping for M7

Aliquots of 50, 100, and 250 U of β-glucuronidase in 150 mM sodium acetate were added to the Phase II reaction sample and incubated at 37 °C for 20 h. The reaction was terminated by the addition of 300 µL 100% ACN, mixed thoroughly and centrifuged at 13,000 rpm for 10 min. A 100 µL aliquot of the supernatant was collected, and 10 µL was injected onto a C18 column for LC-MS/MS analysis. To investigate the UGT isoform associated with glucuronidation from the hydroxylated osthenol, 10 µM osthenol was combined with 5 pmoles human recombinant CYP 1A1 and 1A2 in 0.1 M Kpi buffer (pH 7.4). The reaction was initiated by the addition of NGS solution and incubated for 30 min at 37 °C. Then, 50 µg/mL human recombinant UGT isoforms and 10 mM UDPGA were added and incubated at 37 °C for 30 min. The reaction was terminated by the addition of 400 µL 100% ACN.

### 2.5. Instruments 

The LC-MS/MS system consisted of a Thermo Scientific UHPLC system (Thermo Fisher Scientific, Waltham, MA, USA) equipped with a HPG-3400RS Standard binary pump, WPS 3000 TRS analytical autosampler, and TCC-3000 SD Column compartment. The LC system was coupled with a high-resolution mass spectrometer (Q Exactive™ Focus quadrupole-Orbitrap MS; Thermo Fisher Scientific, Bremen, Germany). A heated electrospray ionization source II (HESI-II) probe was used as an ion generator with nitrogen used as the auxiliary, sheath and sweep gas. The mass spectrometry was operated in negative ion mode, with sheath gas, and the auxiliary gas was set to 45 and 10 aux units, respectively. The other parameters were set as follows: spray voltage to 2.5 KV, capillary temperature to 250 °C, S-lens RF level to 50, and Aux gas heater temperature to 400 °C. The LC method consisted of water (mobile phase A) and ACN (mobile phase B), both of which contained 0.1% formic acid, at a flow rate of 0.24 mL/min at 45 °C. For metabolic profiling, the gradient conditions were as follows: 15–57% of B at 0–21 min, 57–95% of B at 21–23 min, 5% of B at 23–25 min, 95–15% of B at 25–25.1 min, 15% of B at 25.1–30 min. The analytes were separated with a Kinetex^®^ C18 column (150 mm × 2.1 mm, 2.6 μm, Phenomenex, Torrance, CA, USA).

## 3. Results

### 3.1. Identification of Phase I and Phase II Metabolites of Osthenol in HLMs

**Figure 2:**
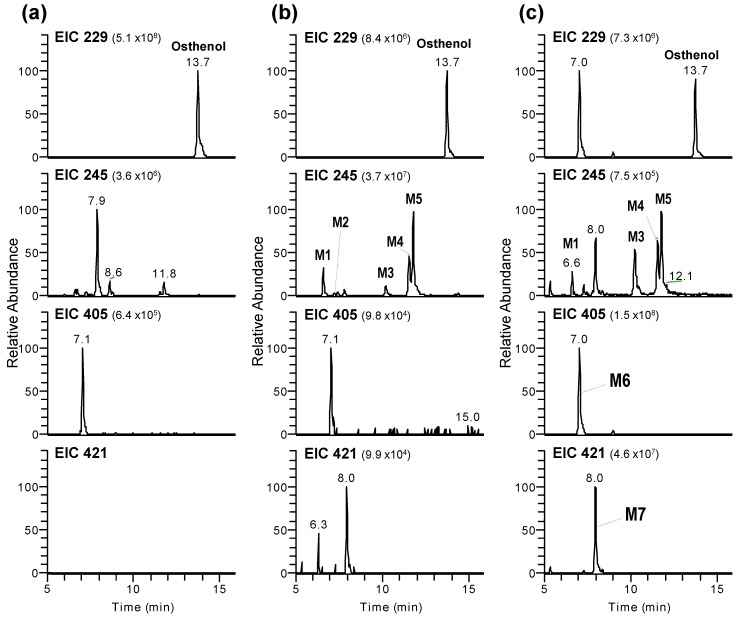
LC-MS/MS analysis of osthenol and its metabolites. Extracted ion chromatograms for osthenol (20 μM) and its metabolites (M1–M7) after 60 min of incubation with 1 mg/mL pooled human liver microsomes in the absence (**a**) and the presence (**b**) of a reduced nicotinamide adenine dinucleotide phosphate (β-NADPH)-regenerating system (NGS), and in the presence NGS with 5 mM uridine 5′-diphosphoglucuronic acid (**c**).

Representative extracted ion chromatograms (EICs) following the incubation of pooled human liver microsomes with osthenol are shown in Figure 2. Osthenol was detected at *m/z* 229 at 13.7 min in negative mode, which showed a stronger intensity compared to the positive mode. After 60 min incubation in the presence of NGS and UDPGA, seven metabolites were generated, and protonated ions in negative ion mode were observed at *m/z* 245 (M1–M5) at 6.6–11.8 min, *m/z* 407 (M6) at 7.0 min and *m/z* 421 (M7) at 8.0 min corresponding to monohydroxylation, *O*-glucuronide conjugate and hydroxyl-glucuronide conjugate, in pooled human liver microsomes, respectively (Table 1). The mass signal of the generated metabolites (M1–M5) in negative ion mode was stronger than in positive ion mode, and M6 and M7 were only detected in negative ion mode. The experiment was carried out through in negative ion mode.

### 3.2. Elucidation of Metabolite Structure

**Figure 3:**
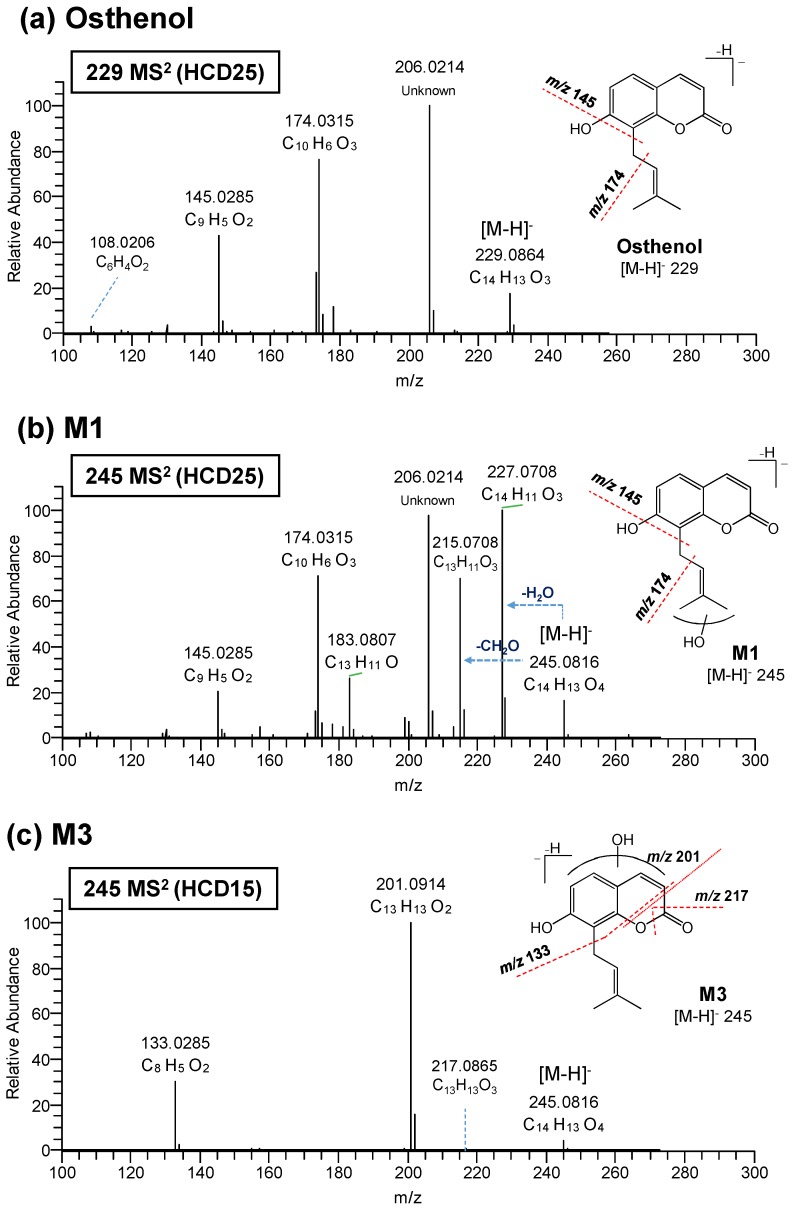

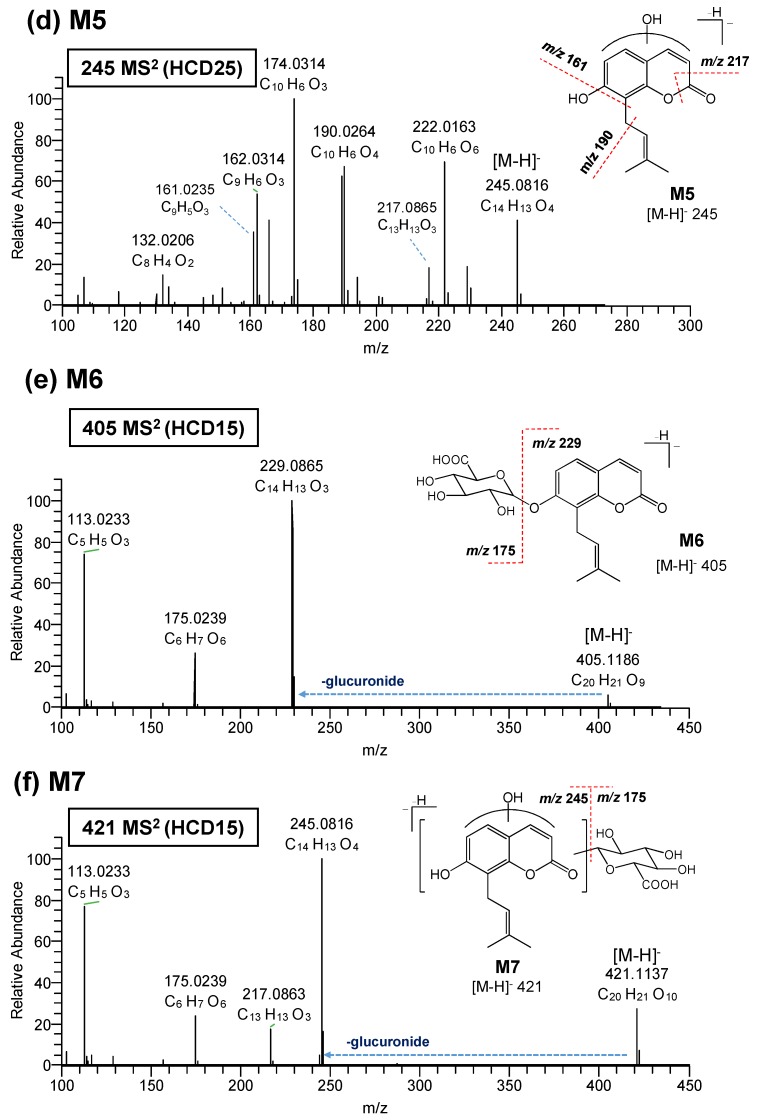
MS/MS spectra of protonated osthenol (**a**), 5 hydroxylated osthenol (M1–M5, (**b**)–(**d**)), osthenol-glucuronide (M6, (**e**)), and hydroxyl glucuronide osthenol (M7, (**f**)) using high-resolution/high-accuracy tandem mass spectrometry.

To elucidate the chemical structure of osthenol metabolites, the HR-MS/MS of osthenol and M1–M7 were characterized using HR-MS/MS. The product ions and fragments were matched to their elemental composition with less than 5 ppm error (Table 1). The product ion spectrum of protonated osthenol is depicted in Figure 3a. The precursor ion spectra of protonated osthenol were observed at [M-H]^−^ 229.0864 (C_14_H_13_O_3_) in negative mode and the dominant fragment ions of MS^2^ were at *m/z* 174.0315 (C_10_H_6_O_3_) and 145.0285 (C_9_H_5_O_2_), indicating the loss of a methylpropene, and methylbutene with hydroxyl group moiety, respectively. Another dominant fragment ion at *m/z* 206.0214 (C_10_H_6_O_5_, loss of 23 Da) was detected, although we could not trace the exact fragment mechanism; it was assumed to indicate 2H-chromen-2-one (coumarin), which would be the key ion to determine the hydroxylation position of metabolites.

The M1 was observed at *m/z* 245.0816 (C_14_H_13_O_4_) as monohydroxylated metabolites in HLMs. The MS^2^ spectrum of M1 shows major product ions at 227.0708, 215.0708, 206.0214, 174.0351, and 145.0285 (Figure 3a). At first, two major product ions at *m/z* 227.0708 (C_14_H_11_O_3_) and 215.0708 (C_13_H_11_O_3_) indicated the loss of H_2_O (18 Da) and CH_2_O (30 Da), respectively. Another three characteristic ions at *m/z* 206.0214 (C_10_H_6_O_5_), 174.0315 (C_10_H_6_O_3_), and 145.0285 (C_9_H_5_O_2_) were the same as those observed in the MS^2^ spectrum of osthenol. Based on these results, these metabolites were proposed to be monohydroxylated on the methyl group in the pentane moiety. The M2 showed the same higher-energy collisional dissociation (HCD) spectra as M1 (Table 1), and was also proposed as a monohydroxylated osthenol in the pentane moiety. 

As depicted in Figure 3c, the MS^2^ spectrum of M3 at *m/z* 245.0816 (C_14_H_13_O_4_) showed a different pattern compared to osthenol and other metabolites. The three product ions detected at *m/z* 217.0865 (C_13_H_13_O_3_), 201.0914 (C_13_H_13_O_2_), and 133.0285 (C_8_H_5_O_2_) indicate the fragmentation in the 2H-chromen-2-one moiety (Table 1). The ion at *m/z* 217 corresponds to loss of -CH_2_O in 5,6-dihydro-2H-pyran-2-one moiety (B ring), and the ion at *m/z* 201 was also the result of being connected to the ion at *m/z* 217. The ion at m/z 133 indicates the loss of a pentane moiety from the ion at *m/z* 201. However, these fragment ions do not indicate the exact position of monohydroxylation. As a result, M3 is postulated as monohydroxyl osthenol in the 2H-chromen-2-one moiety. 

The M4 (*m/z* 245.0817, C_14_H_13_O_4_) and M5 (*m/z* 245.0816, C_14_H_13_O_4_) showed similar MS^2^ fragment patterns (Table 1). The ions at *m/z* 190.0264 (C_10_H_6_O_4_) and 161.0235 (C_9_H_5_O_3_) were observed as newly generated fragment ions compared to osthenol (Figure 3d) and were >16 Da than the corresponding osthenol ions at *m/z* 174 and 145, indicating monohydroxylation in the 2H-chromen-2-one moiety. Although the MS^2^ fragment patterns of M4 and M5 show different patterns compared to M3, they were tentatively assigned as monohydroxyl derivatives at the 2H-chromen-2-one (coumarin moiety). 

In addition, the hydroxylation position of M1–M5 was confirmed by MS/MS fragment in positive ion mode (data not shown). Osthenol showed the specific fragment ion as *m/z* 175 for the loss of 56 Da, which could assume the existence of hydroxylation in the methyl propane moiety. The MS^2^ fragment of M1 and M2 (*m/z* 247.0961) showed the *m/z* 175.0387 (C_10_H_7_O_2_), indicating the loss of hydroxyl methyl propane moiety (72 Da). On the contrary, the MS^2^ fragment of M3-M5 (*m/z* 247.0960) showed the *m/z* 191.0335 (C_10_H_7_O_4_), indicating the loss of the methyl propane moiety (56 Da). Therefore, the structure of the metabolites can be estimated once more through this result.

The precursor ion spectra of the protonated monoglucuronide metabolite M6 of osthenol was observed at *m/z* 405.1186 (C_20_H_21_O_9_) in negative mode (Figure 3e). The fragment ions are listed in Table 1. We confirmed that *m/z* 229.0865 (C_14_H_13_O_3_) indicates the loss of glucuronide moiety from osthenol. The two fragment ions at *m/z* 175.0239 (C_6_H_7_O_6_) and 111.0233 (C_5_H_5_O_3_) indicate the glucuronide moiety. Based on the osthenol structure, M6 is the osthenol-7-*O*-β-D-glucuronide formed in HLMs.

The precursor ion spectra of the protonated monohydroxyl glucuronide metabolite M7 of osthenol was observed at *m/z* 421.1137 (C_20_H_21_O_10_) in negative mode (Figure 3e). We also confirmed that *m/z* 245.0816 (C_14_H_13_O_4_) indicates the loss of the glucuronide moiety from hydroxyl osthenol. The ion at *m/z* 217.0863 (C_14_H_13_O_4_) was newly detected, indicating the loss of -CH_2_O in 5,6-dihydro-2H-pyran-2-one moiety (B ring). The two fragment ions at *m/z* 175.0239 (C_6_H_7_O_6_) and 113.0233 (C_9_H_7_O_2_) were the same as detected in M6. Based on the signature ion at *m/z* 217, M7 was determined to be derived from M3, M4, or M5, which are monohydroxylated osthenol metabolites at the 2H-chromen-2-one (coumarin moiety).

### 3.3. Reaction Phenotyping of Osthenol Metabolism Using cDNA-Expressed Recombinant CYP and UGT Isoforms

**Figure 4:**
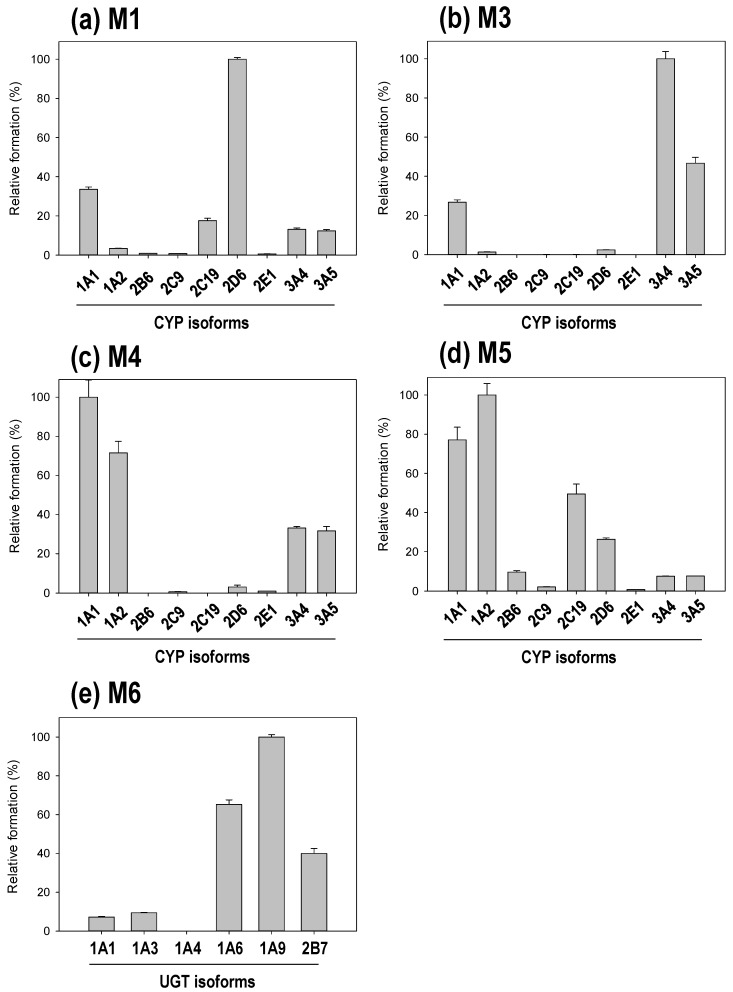
The formation of osthenol metabolites in human recombinant cDNA-expressed cytochrome P450 (CYP) isoforms (**a**–**d**) or cDNA-expressed uridine 5′-diphospho-glucuronosyltransferase (UGT) isoforms (**e**). Osthenol (20 μM) was incubated with each enzyme (5 pmole) for 60 min at 37 °C in the presence of a NADPH-regenerating system and 5 mM uridine 5′-diphosphoglucuronic acid. Data are expressed as the means ± SE of three independent determinations.

For identification of the osthenol metabolic enzymes in HLMs, we incubated osthenol with nine human recombinant CYP (10 pmoles) or six UGT (10 pmoles) isoforms in the presence of NGS or UDPGA, respectively (Figure 4). There are three different groups of CYP isoforms to selectively metabolize to hydroxyl-osthenol. At the first, CYP2D6 mainly generated the formation of M1, monohydroxyl osthenol, in the pentane moiety. While M3 is generated highly by CYP3A4, and another mono-hydroxylated metabolite, 2H-chromen-2-one, M4 and M5 are mainly metabolized by CYP1A1 and 1A2. In addition, CYP2C19 and 2D6 were also partially involved in the formation of M5. Although the structures of M3, M4 and M5 are predicted to be hydroxylation on the 2H-chromen-2-one moiety, their hydroxyl position was presumed to be different because the related enzymes were different, as CYP3A4/3A5 generated M3, and CYP1A1/1A2 contributed to the formation of M4 and M5. M6, the monoglucuronide conjugate, showed the highest formation in incubation with human recombinant UGT1A9, while UGT1A6 was also slightly involved in the formation of M6. 

**Figure 5:**
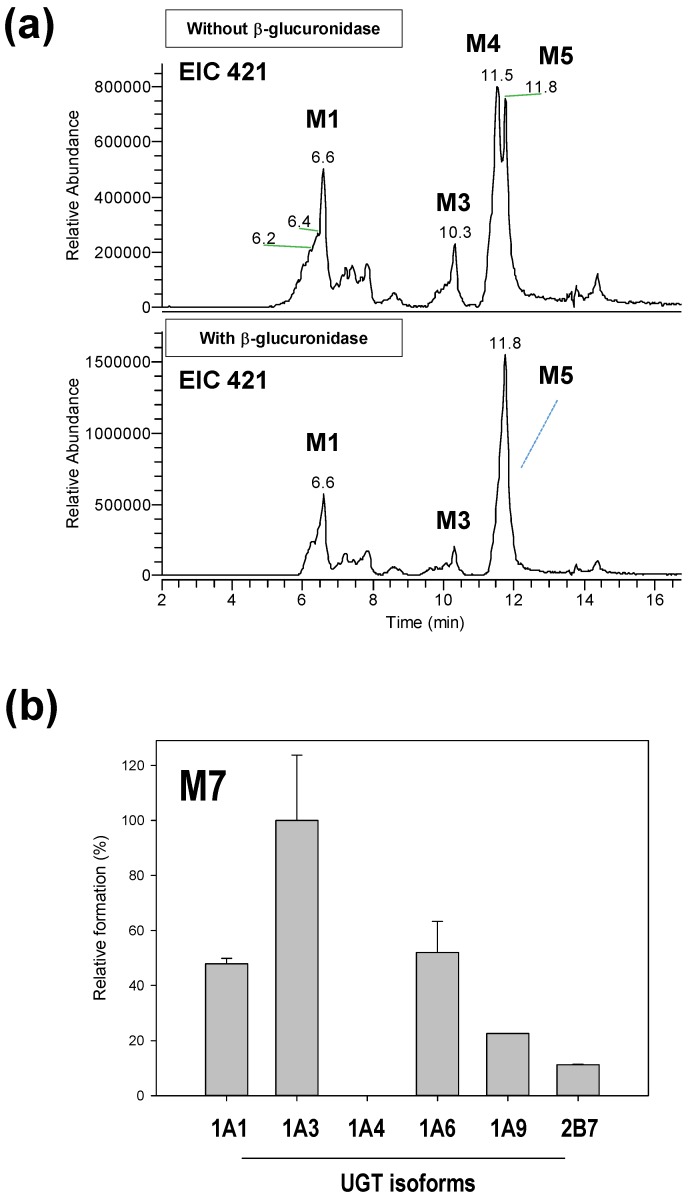
The formation of M7 in human liver microsomes (HLMs). Extracted ion chromatograms for M7 after incubation for 60 min with 1 mg/mL HLMs in the absence and the presence of β-glucuronidase (**a**) and the formation of M7 from M5 in human recombinant cDNA-expressed uridine 5′-diphospho-glucuronosyltransferase (UGT) isoforms (**b**) after treatment with β-glucuronidase.

M7 is assigned as the hydroxyl-glucuronide conjugate of osthenol by the interpretation of MS^2^ analysis (Figure 3f), which is a product of the two step metabolic pathways. In Figure 3f, the M7 is assumed to be derived from the M4 or M5 by the signature ion at *m/z* 217. Moreover, to confirm the enzyme reaction phenotyping for M7, we additionally treated with β-glucuronidase to remove glucuronide from M7 after the osthenol was incubated in presence of NGS and UDPGA in HLMs (Figure 5a). After the β-glucuronidase treatment, M5 increased greatly compared to without β-glucuronidase treatment, which means that M5 is the intermediate for M7 generation. Moreover, to identify the UGT isoform related to M7 conjugation from M5, we incubated it with the human recombinant UGT isoforms UGT1A1, 1A3, 1A4, 1A6, 1A9, and 2B7, after the incubation of osthenol with human recombinant CYP 1A2 (Figure 5b). M7 was mainly generated by UGT1A3, and UGT 1A1 and 1A6 slightly contributed to the formation of M7 from M5.

## 4. Discussion

**Figure 6:**
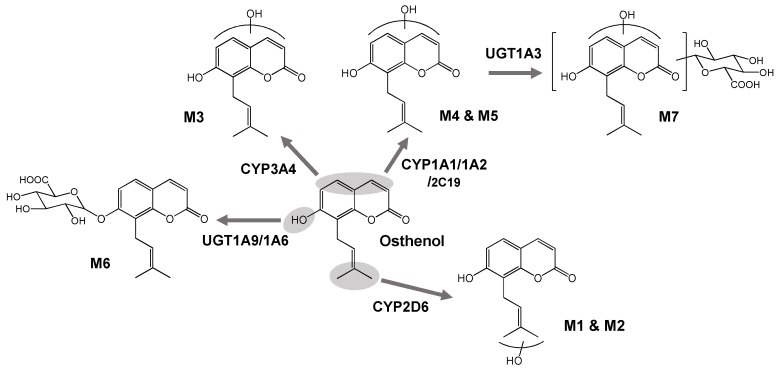
Postulated metabolic pathways of osthenol in human liver microsomes.

Osthenol is a prenylated coumarin, and is known to be a major metabolite of osthole, desmethyl-osthole [9,10]. Although osthole is known to be the major component of the bioactive reaction of Cnidii Fructus [8], osthenol also shows bioactivation, such as the inhibition of skin tumor promotion [4], strong antibacterial and antifungal activities [5,6] and anti-inflammatory effects [7]. To date, the metabolite directly generated from osthenol has not been identified, and the metabolic enzymes involved in osthenol metabolism remain unknown. Here, we investigated the metabolic pathway of osthenol in HLMs and identified five hydroxylated metabolites by CYPs, and two glucuronide conjugates of osthenol by UGTs (Figure 6).

Previously, the metabolic pathway of osthenol was partially identified in a study of osthole in rats. In 2013, the metabolites of osthole, 7-*O*-methyl-osthenol, were isolated from rat urine, and the structures of 10 phase I and three phase II metabolites were identified using the 2D-NMR technique [10]. Among these metabolites, osthenol-related metabolites were identified, including 5′-hydroxyl-osthenol, 4′-hydroxyl-2′, 3′-dihydro-osthenol, 4′-hydroxyl-osthenol for phase I, and osthenol-7-*O*-β-D-glucuronide for phase II. In addition, Li et al. studied the metabolism of osthole in rat urine by UPLC-QTOF/MS in 2013 [12]. They identified a total of 11 metabolites by 7-demethylation, 8-dehydrogenation, hydroxylation on coumarin, 3,4-epoxide, and sulfate conjugation.

Based on these studies, we expected that the metabolic pathway of osthenol would show it to be monohydroxylated at the 4′C or 5′C position, and between 3C to 6C in 2H-chromen-2-one, with dehydrogenation at 2′-3′C for phase I, and 7-*O*-glucuronidation and 7-*O*-sulfation for phase II. First, we identified five monohydroxylated metabolites that could be distinguished by three distinct features (Figure 6). M1 and M2 are monohydroxylated metabolites at 4′C or 5′C and are metabolized by CYP2D6. The typical substrate of CYP2D6 usually consists of lipophilic bases with a planar hydrophobic aromatic ring and a nitrogen atom, however CYP2D6 can also react with several atypical substrates that do not contain a basic nitrogen atom [13]. M3 is produced by monohydroxylation on a 2H-chromen-2-one moiety by CYP3A4 and CYP3A5, which prefer relatively large, structurally diverse molecules [14]. M4 and M5 are also monohydroxylated metabolites of a 2H-chromen-2-one moiety; the hydroxylation positions of M4 and M5 are expected to be different from those of M3. Although M5 was generated by CYP2C19 and 2D6, CYP1A1 and 1A2 are the main isoforms that generate M4 and M5 in HLMs. Planar molecules, neutral or basic in character, are typical substrates of CYP1A2 [14]. Although the dehydrogenated metabolite of osthenol was reported in rat urine and microsomal enzymes can mediate dehydrogenation, this metabolite was not detected in the present study.

In natural phenolic phytochemicals, glucuronidation is linked to significant physiological properties, such as its solubility, bioactivity, bioavailability, and inter- and intracellular transport [15]. Moreover, extensive glucuronidation can be a barrier to oral bioavailability as the first-pass glucuronidation of orally administered agents usually results in poor oral bioavailability and a lack of efficacy [16]. In the present study, osthenol and hydroxyl-osthenol (M5) were strongly glucuronidated by UGT in HLMs, and M6, 7-*O*-glucuronide-osthenol, was produced 200-fold more than hydroxyl osthenol in the presence of NGS and UDPGA (Figure 2c). The hydroxylated metabolite (M5) of osthenol was also strongly converted to glucuronide conjugate (M7), and the concentration of hydroxylated metabolites decreased, indicating that the glucuronidation of osthenol is major metabolic pathway and controls the pharmacokinetic parameters of osthenol in vivo. Here UGT1A6 and 1A9 mainly contributed to the 7-*O*-glucuronidation of osthenol, which is consistent with previous studies of the glucuronidation of coumarins. For example, 4-methylumbelliferone is metabolized by human UGT1A6, 1A7, and 1A10 [17]. Human UGT1A6 and 1A9 were shown to be major isoforms involved in daphnetin glucuronidation in human intestine and liver microsomes [18]. Scopoletin is rapidly metabolized by UGT1A3, 1A6, and 1A9 [19]. In addition, UGT1A3 is dominantly associated with M7 generation from M5, where coumarin moiety is glucuronidated.

In conclusion, we demonstrated that osthenol is metabolized to five monohydroxyl metabolites (M1–M5) by CYP2C19, 2D6, 1A2, and 3A4, respectively, and a 7-*O*-glucuronide conjugate (M6) by UGT1A9 and a hydroxyl-glucuronide (M7) by UGT1A3 in HLMs. We also found that glucuronidation is a dominant metabolic pathway of osthenol in HLMs. The structures of the metabolites were proposed using high-resolution/high-accuracy tandem mass spectrometry, and the possible metabolic fate of osthenol in HLMs is summarized in Figure 6.

## Figures and Tables

**Table 1:** Elemental composition of key product ions of osthenol and its metabolites in human liver microsomes using high-resolution quadrupole-orbitrap mass spectrometry.

Compound	Precursor Ions (*m/z*)			HCD (eV)	Product Ion (*m/z*)	Elemental Comp. (exp.)	Error (ppm)	Mass Shift (Da)
	MS^2^	Elemental Comp. (exp.)	Error (ppm)					
Osthenol	229.0864	C_14_H_13_O_3_	−0.5	25	206.0214	Unknown	NA	
				25	174.0315	C_10_H_6_O_3_	−1.1	
				25	145.0285	C_9_H_5_O_2_	−3.2	
				25	130.0416	C_9_H_6_O	−1.8	
				25	108.0206	C_6_H_4_O_2_	−5.0	
M1	245.0816	C_14_H_13_O_4_	1.0	25	227.0708	C_14_H_11_O_3_	−0.1	-H_2_O
				25	215.0708	C_13_H_11_O_3_	−0.1	-CH_2_O
				25	206.0214	Unknown	NA	
				25	183.0807	C_13_H_11_O	−1.4	
				25	174.0315	C_10_H_6_O_3_	−1.3	
				25	145.0285	C_9_H_5_O_2_	−2.9	
				25	108.0207	C_6_H_4_O_2_	−4.2	
M2	245.0816	C_14_H_13_O_4_	2.9	25	227.0708	C_14_H_11_O_3_	2.7	-H_2_O
				25	215.0707	C_13_H_11_O_3_	2.2	
				25	206.0214	Unknown	NA	
				25	183.0808	C_13_H_11_O	1.9	
				25	174.0314	C_10_H_6_O_3_	1.5	
				25	145.0285	C_9_H_5_O_2_	0.9	
M3	245.0816	C_14_H_13_O_4_	3.2	15	217.0865	C_13_H_13_O_3_	2.7	
				15	201.0914	C_13_H_13_O_2_	2.0	
				15	133.0285	C_8_H_5_O_2_	0.5	
M4	245.0817	C_14_H_13_O_4_	3.4	25	229.0501	C_13_H_9_O_4_	2.5	
				25	222.0164	C_10_H_6_O_6_	2.3	206 + 16
				25	201.0914	C_13_H_13_O_2_	1.9	
				25	190.0264	C_10_H_6_O_4_	1.8	174 + 16
				25	173.0965	C_12_H_13_O	2.4	
				25	162.0314	C_9_H_6_O_3_	1.4	
				25	132.0207	C_8_H_4_O_2_	0.9	
M5	245.0816	C_14_H_13_O_4_	1.0	25	229.0501	C_13_H_9_O_4_	0.2	213 + 16
				25	222.0163	C_10_H_6_O_6_	−0.4	206 + 16
				25	217.0865	C_13_H_13_O_3_	0.4	
				25	190.0264	C_10_H_6_O_4_	−1.4	174 + 16
				25	174.0314	C_10_H_6_O_3_	−1.6	
				25	166.0262	C_8_H_6_O_4_	−2.3	
				25	162.0314	C_9_H_6_O_3_	−1.5	
				25	161.0235	C_9_H_5_O_3_	−2.6	145 + 16
				25	132.0206	C_8_H_4_O_2_	−3.7	
M6	405.1186	C_20_H_21_O_9_	1.5	15	229.0865	C_14_H_13_O_3_	2.7	
				15	175.0239	C_6_H_7_O_6_	1.3	
				15	113.0233	C_5_H_5_O_3_	−0.1	
M7	421.1137	C_20_H_21_O_10_	1.8	15	245.0816	C_14_H_13_O_4_	3.1	
				15	217.0863	C_13_H_13_O_3_	1.8	
				15	175.0239	C_6_H_7_O_6_	1.3	
				15	113.2333	C_5_H_5_O_3_	−0.2	
